# Caregiver education for childhood respiratory conditions in Africa

**DOI:** 10.7196/AJTCCM.2018.v24i2.215

**Published:** 2018-06-21

**Authors:** Robin J Green

**Affiliations:** Department of Paediatrics and Child Health, Faculty of Health Sciences, University of Pretoria, Pretoria, South Africa

## Editorial

The world has started to realise that both acute and chronic medical conditions are managed best when caregivers (parents and teachers) know something about the condition of the children in their care.^[1]^ Even in developed nations, this process is limited by apathy on the part of medical personnel; in Africa, there is real concern that educational strategies for many conditions are seriously neglected. This problem may arise in part from the lack of government funding for educational programmes, and in part because many respiratory conditions are neglected, because management of HIV and tuberculosis (TB) has enjoyed greater emphasis in medical programmes.

Asthma is a common chronic illness affecting children. Despite some educational programmes for management of chronic asthma being in place, very few stress the principles of managing acute exacerbations. In addition, students spend a considerable amount of time in school, and asthma control may be influenced by the knowledge of the teachers, and the facilities available to support them.

In this issue of the *AJTCCM*, Adeyeye *et al*.^[2]^ report on their study that assessed the knowledge of secondary school teachers in Lagos, Nigeria, regarding asthma, and evaluated the facilities and personnel available in the schools to support asthmatic students during emergencies.

Of the 988 teachers sampled in this study, 475 (48.1%) had poor knowledge of managing asthma, 414 (41.9%) had fair knowledge and only 99 (10%) had what the authors describe as ‘good’ knowledge. None of the schools had a nebuliser available for treatment of an asthma emergency. The authors conclude that teachers in secondary schools in Lagos have unsatisfactory knowledge of managing asthma exacerbations.

I would not be surprised if this finding were also to apply elsewhere in Africa. The response may be better in South Africa (SA), where the National Asthma Education Programme has had input into school care for asthmatics, and the Allergy Society of SA has been in dialogue with school governing bodies with regard to the management of food allergies and anaphylaxis.^[3,4]^ However, even in SA, this approach would not be available throughout the country.

We need to learn, and learn fast, from our colleagues who manage HIV infection and TB. Those management programmes have had much success in promoting understanding, drug adherence and reporting of symptoms.

We cannot stand by while children die of respiratory conditions such as asthma. These conditions are important in Africa.
